# The gut microbiota as a target to improve health conditions in a confined environment

**DOI:** 10.3389/fmicb.2022.1067756

**Published:** 2022-12-19

**Authors:** Zheng Chen, ZiYing Wang, Dan Li, Beiwei Zhu, Yongjun Xia, Guangqiang Wang, Lianzhong Ai, Chunhong Zhang, Chuan Wang

**Affiliations:** ^1^School of Food Science and Technology, Dalian Polytechnic University, Dalian, China; ^2^Navy Special Medical Center, Naval Medical University, Shanghai, China; ^3^School of Health Science and Engineering, Shanghai Engineering Research Center of Food Microbiology, University of Shanghai for Science and Technology, Shanghai, China

**Keywords:** confined environment, gut microbiota, health, uric acid, urine chemical index

## Abstract

Confined environments increase psychological stress and lead to health problems such as abnormal mood and rhythm disruption. However, the mechanism by which confined environments impact health has remained unclear. Significant correlations have been reported between psychological stress and changes in gut microbiota. Therefore, we investigated the effect of a confined environment on the composition of the gut microbiota by 16s rDNA high-throughput sequencing, and analyzed the correlation between gut microbiota and health indicators such as uric acid (UA), sleep, and mood. We found that the gut microbiota of the subjects clustered into two enterotypes (Bi and Bla), and that the groups differed significantly. There were notable differences in the abundances of genera such as *Bifidobacterium*, *Dorea*, *Ruminococcus_torques_group*, *Ruminococcus_gnavus_group*, *Klebsiella*, and UCG-002 (*p* < 0.05). A confined environment significantly impacted the subjects’ health indicators. We also observed differences in how the subjects of the two enterotypes adapted to the confined environment. The Bi group showed no significant differences in health indicators before and after confinement; however, the Bla group experienced several health problems after confinement, such as increased UA, anxiety, and constipation, and lack of sleep. Redundancy analysis (RDA) showed that UA, RBC, mood, and other health problems were significantly correlated with the structure of the gut microbiota. We concluded that genera such as UCG-002, *Ruminococcus*, CAG352, and *Ruminococcus_torques_group* increased vulnerability to confined environments, resulting in abnormal health conditions. We found that the differences in the adaptability of individuals to confined environments were closely related to the composition of their gut microbiota.

## Introduction

In environments such as those required for aerospace travel, voyages, and home isolation, individuals reside in small and relatively closed spaces for long periods of time; these periods are also often accompanied by information isolation (Li et al., 2016; LaGoy et al., 2020; Nie et al., 2021). Confined environments (CEs) can gradually increase psychological stress, resulting in functional gastrointestinal disorders, depression, irritability, and other health problems (Holmes and Simmons, 2009; Paul et al., 2010; Bell et al., 2019). The working conditions and environmental microorganisms in CEs can have additional effects on psychological and physical health (Berg et al., 2005; Reini, 2010; Kimhi, 2011). The temperature, humidity, and gaseous composition of CEs can also affect the physiological and cognitive well-being of the staff (Li et al., 2018).

Studies have shown that continuous increases in psychological stress can lead to abnormal moods and affect gastrointestinal function and lipid metabolism homeostasis through bidirectional regulation of the gut-brain axis; the gut microbiota play a very important role in this process (Arase et al., 2016). Multiple complex interactions have been recorded between psychological stress and gut microbiota. Indeed, disturbances to gut microbiota have been documented to promote disease progression (Xu et al., 2020). Allen et al. (2021) showed that psychological stress can trigger intestinal epithelial cells to express pro-inflammatory molecules and promote reactive oxygen species (ROS) production. This expression is stimulated by the presence of gut microbiota, the composition of which can gradually change in response to ROS stress. The generation of intestinal ROS is related to physiological activities such as mood changes and insomnia (Brainard et al., 2016; Palinkas and Suedfeld, 2021). Studies have shown that intestinal cells secrete uric acid (UA) upon hydrogen peroxide stimulation to resist oxidative stress and that the increase in UA is related to imbalances in the gut microbiota (Wang et al., 2022).

Some CE-simulation experiments show that light, gas, and work rhythms significantly affect the psychological states of the subjects, in turn affecting their cognitive abilities, teamwork, and other aspects of work efficiency (Berg et al., 2005; Kimhi, 2011). However, changes in gut microbiota in CEs and how they correlate with health indicators have remained unclear. Although current research on gut microbiota has focused on the association of gut microbiota with physical and chemical indicators of disease, few studies have investigated the influence of gut microbiota on urine metabolism. Therefore, we performed 16 s rDNA sequencing of the gut microbiota of subjects in a CE to analyze changes in the gut microbiota composition. We then investigated the correlations between gut microbiota and urine metabolism, sleep, mood, and other health conditions and screened the relevant landmark flora. This study therefore lays the foundation for examining the impact of CEs on human health.

## Materials and methods

### Subject

The subjects were 12 healthy males, aged 19–26 years, 163–186 cm tall and 54–95 Kg in weight. The subjects did not have cardiovascular system diseases, hemorrhoids, infectious diseases and skin diseases, etc., did not take alcohol, antibiotics and other drugs 7 days before the experiments, and maintained a good sleep. Before participating in the experiment, the subjects have understood the process and risks of this experiment, and signed the informed consent form. All experiments were approved and performed following the guidelines of the Ethical Committee of Naval Medical University (Approval No. AF-HEC-018).

### Confined environment

**Figure 1:**
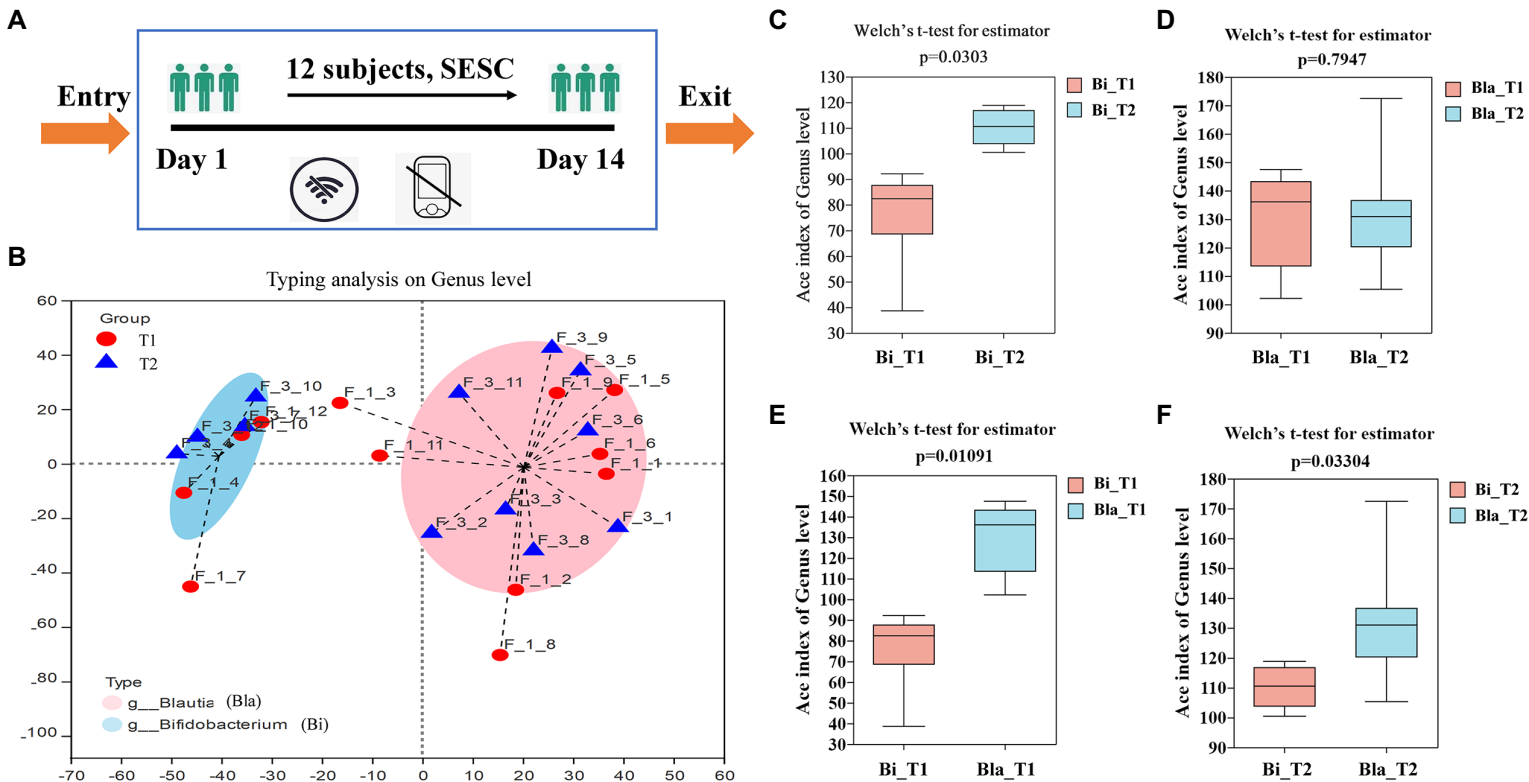
Enterotypes analysis and α-diversity changes of gut microbiota in confined environment. **(A)** The flow chart of experimental; **(B)** Enterotypes analysis of gut microbiota (day 1, T1; day 14, T2); **(C–F)** The α-diversity changes of gut microbiota in different groups. Bla group, containing subjects of 4, 7, 10, 12. Bi group, containing subjects of 1, 2, 3, 5, 6, 8, 9, 11. Sample F_1_1 means subject 1 in day 1. Sample F_3_1 means subject 1 in day 14. Results are express as the mean ± SEM of subjects for each experimental group (Bi_T1 = 4, Bi_T2 = 4, Bla_T1 = 8, Bla_T2 = 8).

The experiment was carried out in submarine environment simulation cabin (SESC). The living conditions are normal pressure and room temperature in the confined environment, and the effective volume is about 200 m^3^. The errors of temperature and relative humidity in the room are less than ±0.5°C and ± 5 %RH. During the experiment, the subjects were forbidden to smoke and drink, eat normally every day. The subjects could use computers, fitness equipment, etc., but there was no external network, and the submarine environment simulation cabin was in a state of information isolation. The entire experiment lasted 14 days and nights. The flow chart of experimental was shown in Figure 1A.

### Sample collection

Faecal samples of subjects were collected at 18:00–22:00 on day 1 and day 14. The faecal samples of one subject were added into sterilized 5-mL Eppendorf tubes on ice and stored at −80°C. Urine samples of subjects were collected at 7:30 am on day 1 and day 14. The Urine samples of one subject were added into 20-mL sampling tube on ice and stored at −80°C.

### 16S rRNA gene sequencing of gut microbiota

Faecal samples of subjects were prepare for microbiota analysis. Faecal DNA was extracted using an E.Z.N.A. stool DNA Kit (Omega Bio-tek, USA) according to the manufacturer’s instructions. The V3-V4 regions of the bacterial 16S rRNA genes were amplified using universal primers 338F 5′-ACTCCTACGGGAGGCAGCA-3 and 806R 5′-GGACTACHVGGGTWTCTAAT-3′. PCR amplification of the 16S rRNA gene was carried out in triplicate as follow: template DNA 10 ng, 2.5 mM d NTPs 2 μl, forward and reverse primer (5 μM) 0.8 μl respectively, TransStart fast Pfu polymerase 0.4 μl, 5 × Fast Pfu Buffer 4 μl, and ddH_2_O in a final volume of 20 μl. PCR amplification program was as follows: an initial activation step with 95°C for 3 min, followed by 27 cycles at 95°C for 30 s, 55°C for 30 s, and 72°C for 30 s and a final extension at 72°C for 10 min (Dennis et al., 2013). The quality of PCR products was quantified using QuantiFluor™-ST system (Promega, USA) according to the standard protocols. Then, purified PCR products were sequenced on an Illumina MiSeq platform (Illumina, USA) at Majorbio Bio-Pharm Technology Co., Ltd., Shanghai, China.

### Microbiota analysis

Microbiota analysis of subjects were performed by Majorbio Cloud^1^ provided by Majorbio Bio-Pharm Technology Co. Ltd. (Shanghai, China). All raw reads were demultiplexed and quality-filtered using QIIME (version 1.9.1) with the following criteria: (1) The 300 bp reads were truncated at any site receiving an average quality score < 20 over a 10 bp sliding window abandoned the truncated reads that were < 50 bp; (2) Sequences that reads containing ambiguous characters, or two nucleotide mismatches in primer matching were removed. An operational taxonomic units (OTUs) were set as at least 97% identified sequences. The invalid sequences were cut-off using UPARSE (version 11^2^). RDP Classifier (version 2.13^3^) was used to analyses the taxonomy of each 16S rRNA gene sequence, and against the SILVA (version 138) 16S rRNA database using a confidence threshold of 70% (Quast et al., 2013).

### Determination of urine chemical index

The RBC counting in urine was determined by automated urine analysis workstation IQ200 (Beckman Coulter, Brea, California, America). The UA content in urine was determined by automatic biochemical analyser Cobas C702 (Roche, Basel, Swiss).

### Health conditions survey

The subjects of health conditions were investigated with a questionnaire described by Li et al. (2016). The questionnaire was carried out before and after the experiment, including sleep quality and time, constipation, anxiety, memory loss, dizziness, anorexia, and facial acne. In addition to sleep time, each other health problem is scored as 5 points, and the total score of each item after accumulation was used to represent the health status of the subject.

### Statistical analysis

The α-diversity analysis of different groups was detected by Welch’s t test. Differential relative abundance of taxa was detected by Kruskal-Wallis-H test with corrected *p* value, and the multiple test correction was fdr. Linear regression of gut microbiota based on Bray_Curtis distance was performed at the genus level to assess the impact of health indicators on gut microbiota. Enterotypes analysis of gut microbiota based on JDS distance was performed at the genus level. LEfSe analysis of gut microbiota was performed by using multi group comparison strategy: All gain all (more strict). The correlations between the relative abundance of species and health indicators were calculated by Spearman’s rank correlation coefficient and visualized by heatmap. Statistical analysis of physical and chemical indexes of urine was performed with ANOVA (SPSS 24.0). A *p*-value of <0.05, 0.01 or 0.001 was considered statistically significant.

## Results

### Enterotypes analysis of gut microbiota in a CE

16 s rDNA high-throughput sequencing was used to detect the gut microbiota composition of the subjects during the CE experiment. We obtained 1,177,407 sequences, which were clustered into 641 operational taxonomic units comprising 12 phyla, 89 families, and 233 genera. Based on the abundance of flora at the genus level, the Jensen-Shannon distance was calculated, and clustering was performed by partitioning around medodids to obtain the best clustering K value. Principal coordinate analysis was used to determine the enterotype of the gut microbiota (Wu et al., 2011). As shown in Figure 1B, the gut microbiota enterotype analysis showed that the 12 subjects could be clustered into two enterotypes. Type 1 was the *Blautia* type (Bla, comprising subjects 4, 7, 10, and 12), and type 2 was the *Bifidobacterium* type (Bi, comprising subjects 1, 2, 3, 5, 6, 8, 9, and 11). The subjects of both groups showed a high degree of overlap in terms of gut microbiota type, but the flora characteristics were significantly different. Therefore, subsequent gut microbiota analysis was carried out based on the enterotype. α-diversity analysis of the enterotype groups showed that the α-diversity of the Bi group significantly increased in the CE (*p* < 0.05), whereas that of the Bla group did not significantly change (Figures 1C,D). Horizontal comparison showed that there were significant differences in the α-diversity of the gut microbiota of the two enterotype groups during the experimental process, which was consistent with the results of the subjects’ gut microbiota typing (Figures 1E,F).

### Effect of CE on the gut microbiota of different enterotypes

**Figure 2:**
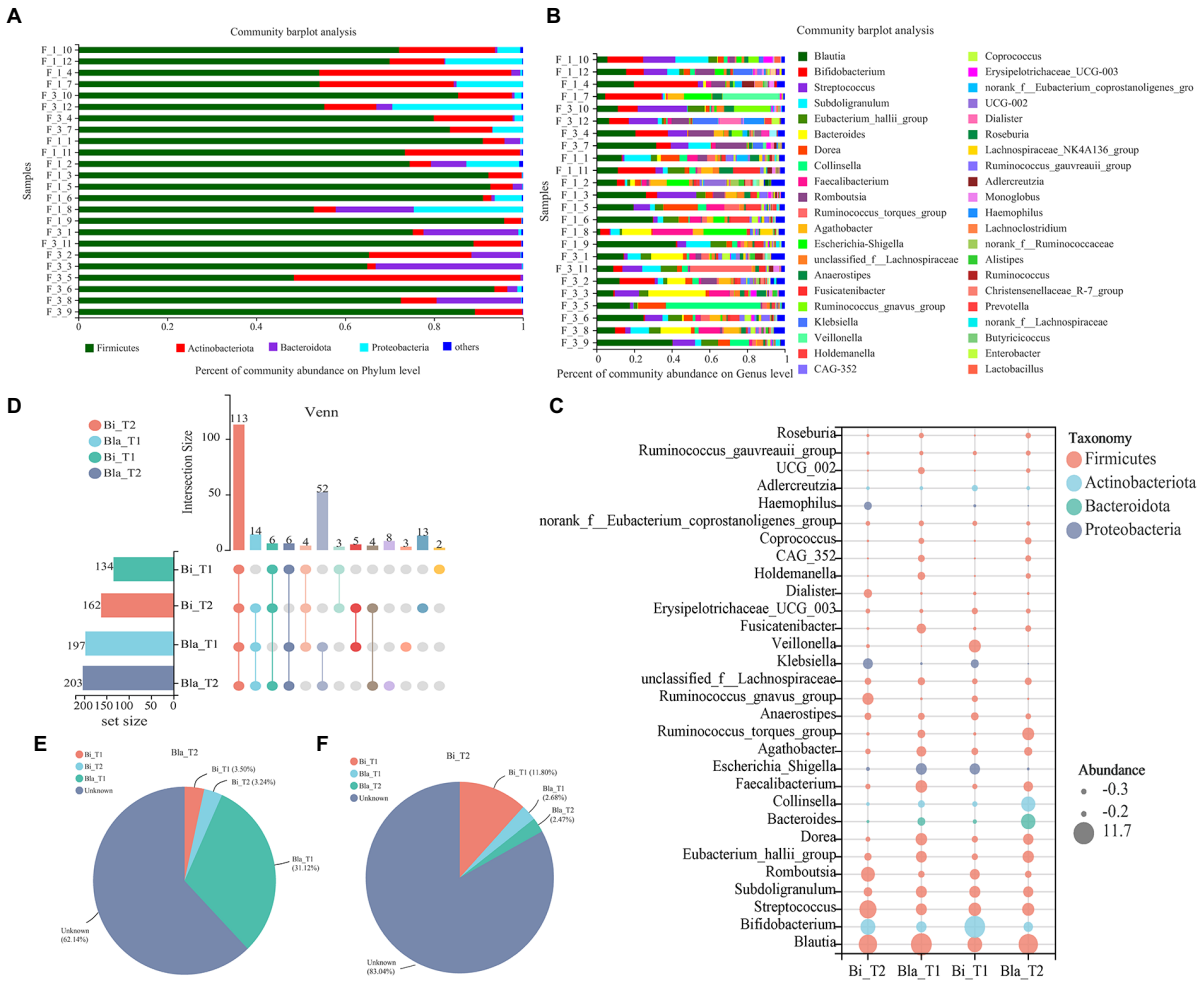
Changes in gut microbiota composition in confined environments. **(A)** Community bar-plot analysis of the relative abundance of gut microbiota on Phylum level. **(B)** Community bar-plot analysis of the relative abundance of gut microbiota on Genus level. **(C)** Bubble plot of gut microbiota in different enterotype groups. Bubble size represents the abundance of gut microbiota. **(D)** UpSet Venn diagram of gut microbiota in different enterotype groups. **(E)** Source tracker analysis of gut microbiota in Bla enterotype group. **(F)** Source tracker analysis of gut microbiota in Bi enterotype group.

As shown in Figure 2, the effect of the CE on the gut microbiota of different enterotypes was analyzed at the phylum and genus levels. The gut microbiota of subjects in the Bi enterotype group were dominated by *Firmicutes*, *Actinobacteriota*, and *Proteobacteria*; these three phyla accounted for >99% of the total abundance, with *Firmicutes* and *Actinobacteriota* amounting to 54 and 37%, respectively. In the Bla group, *Firmicutes*, *Actinobacteriota*, and *Bacteroidota* were the main phyla and accounted for >99% of the total population; *Firmicutes* and *Bacteroidota* amounted to 41 and 25%, respectively (Figure 2A). At the genus level, *Blautia*, *Bifidobacterium*, *Streptococcus*, and *Subdoligranulum* were the main components in the Bla group. However, compared with the Bla group, *Bifidobacterium* was more abundant in the Bi group (Figure 2B). The composition of the gut microbiota was further analyzed using a bubble abundance map (Figure 2C), which showed the top 30 most abundant genera; the sizes of the circles represented differences in the relative abundances of the gut microbiota. Most of the gut microbiota belonged to the phylum *Firmicutes*, with the compositions of the Bla and Bi groups being significantly different. The predominant genera in the two groups were different, with *Bifidobacterium*, *Collinsella*, and other bacteria belonging to the *Actinobacteriota* phyla being higher in the Bi group than in the Bla group.

Figure 2D shows a Venn analysis of gut microbiota in the CE. We found 113 core microbiota species in common across the four groups, with the number of microbiotas in the Bi group being less than that in the Bla group at the genus level. After 14 days of the CE experiment, the number of microbiotas in the Bi group increased; however, there was no significant change in the Bla group. Source Tracker analysis showed that 31.12% of the microbiota in the Bi_T2 group came from the Bi_T1 group. However, the origins of the majority of the microbiota (62.14%) were unclear, which may reflect the effect of the CE (Figure 2E). Only 11.8% of the microbiota in the Bi_T2 group was derived from Bi_T1, and the origins of most of the microbiota (83.04%) remained unclear (Figure 2F).

### Difference in the gut microbiota of enterotypes in response to the CE

**Figure 3:**
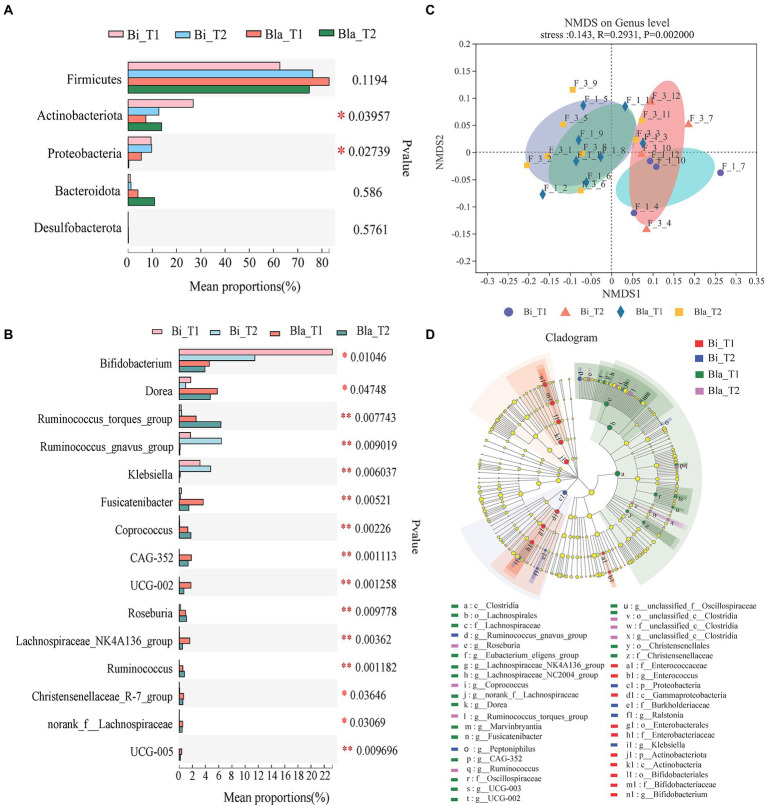
Analysis of differences in gut microbiota of subjects in confined environment. **(A)** Phylotypes significantly different between Bi_T1, Bi_T2, Bla_T1 and Bla_T2 group on Phylum level. **(B)** Phylotypes significantly different between Bi_T1, Bi_T2, Bla_T1 and Bla_T2 group on Genus level. **(C)** NMDS (Non-metric multidimensional scaling) analysis of gut microbiota. **(D)** LEfSe (Linear discriminant analysis effect size) analysis of gut microbiota. LDA scores (threshold >2.0) as calculated by LEfSe of taxa differentially abundant in Bi and Bla groups during the experiments (T1 to T2). The diameter of each circle was proportional to the relative abundance of taxa. Results are express as the mean ± SEM of subjects for each experimental group (Bi_T1 = 4, Bi_T2 = 4, Bla_T1 = 8, Bla_T2 = 8), *p < 0.05, **p < 0.01.

There were significant differences in the gut microbiota of the two enterotypes in the CE (Figure 3). Differences in the dominant phyla and genera (Figures 3A,B) of the gut microbiota were analyzed. At the phylum level, there were significant changes in *Actinobacteriota* and *Proteobacteria* across the four groups (*p* < 0.05). Analysis at the genus level showed that in the Bi group, the relative abundances of *Bifidobacterium*, *Ruminococcus_gnavus_group*, and *Klebsiella* were significantly higher than in the Bla group (*p <* 0.05, *p* < 0.01). However, the abundances of *Dorea*, *Ruminococcus_torques_group*, *Fusicatenibacter*, *Coprococcus*, CAG-352, UCG-002, and other genera were significantly lower in the Bi group than in the Bla group (*p* < 0.05, *p* < 0.01). We performed non-metric multidimensional scaling (NMDS) analysis of the gut microbiota of the subjects and verified the differences in the compositions of the Bi and Bla groups in the CE. After living in the CE for 14 days, the composition of the gut microbiota of the Bi group underwent significant changes, whereas that of the Bla group experienced only small changes (Figure 3C). As shown in Figure 3D, linear discriminant analysis (LEfsE; linear discriminant analysis threshold = 2) showed that *Bifidobacterium*, *Enterococcus*, and other genera were significantly enriched in the Bi_T1 group, and that *Klebsiella*, *Ruminococcus_gnavus_group*, and other genera were significantly enriched in the Bi_T2 group. *Dorea*, *Fusicatenibacter*, UCG-002, and other genera were significantly enriched in the Bla_T1 group, and *Ruminococcus* and *Ruminococcus_torques_group* were significantly enriched in the Bla_T2 group.

**Figure 4:**
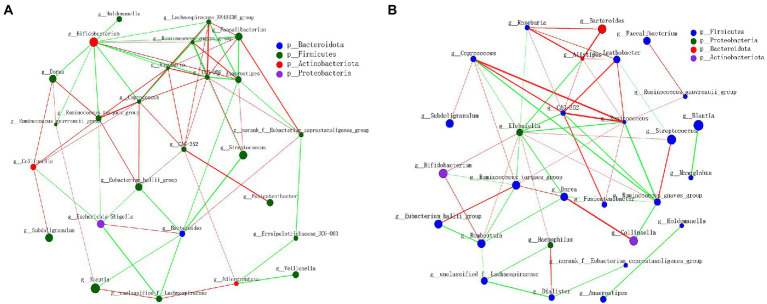
Network analysis applied to the gut microbiota of subjects in confined environment. **(A)** At the beginning of experiment (day 1, T1); **(B)** At the end of experiment (day 14, T2). The size of the node indicates the abundance of genera, and the node color corresponds to phylum taxonomic classification. The color of the line represents positive (green) and negative (red) correlations, and the thickness of the line is equivalent to the correlation values.

The relationships between the gut microbiota of subjects in a CE were investigated through correlation network analysis. Genus-level correlation network analysis revealed significant interactions between the different genera of gut microbiota (a green connection line represents a positive correlation, and red represents a negative correlation; the thickness of the line represents the magnitude of the correlation coefficient; Figure 4). We documented close interactions between *CAG-352, Coprococcus, Ruminococcus*, and other genera, with the relationship becoming stronger after the CE experiments.

### Effects of CE on urine physicochemistry and health indicators of subjects

**Figure 5:**
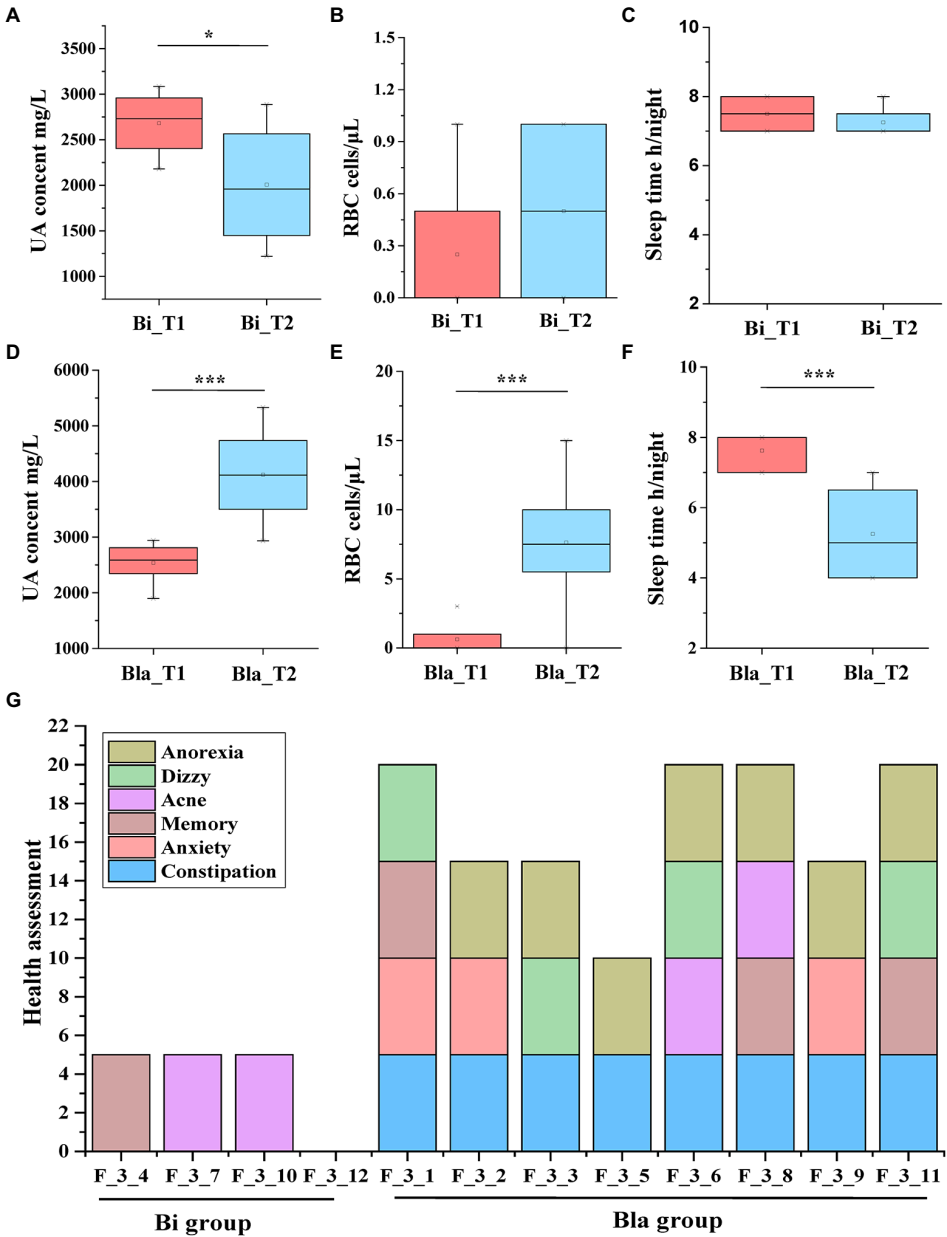
Changing in UA, RBC and other health indicators of subjects in confined environment. **(A)** UA content of urine in Bi group; **(B)** RBC counting of urine in Bi group; **(C)** Sleep time of subjects in Bi group; **(D)** UA content of urine in Bla group; **(E)** RBC counting of urine in Bla group; **(F)** Sleep time of subjects in Bla group; **(G)** Health assessment of subjects at the end of experiment (day 14). Results are express as the mean ± SEM of subjects for each experimental group (Bi_T1 = 4, Bi_T2 = 4, Bla_T1 = 8, Bla_T2 = 8), *p < 0.05, ***p < 0.001.

The urine physicochemistry and health indicators of the subjects during the CE experiment were evaluated. As shown in Figure 5, there were significant differences in the urine physicochemical (UA content, red blood cell (RBC) content, sleep time (S), and other health indicators (H)) between the Bi and Bla groups after living in a CE. There was no difference in other physicochemistry indexes in the urine of the subjects during the experiment (Supplementary Table 1). In the Bi group, the UA content decreased significantly after 14 days in the CE (*p* < 0.05; Figure 5A). There was no significant difference in the RBC content and S between the Bi_T1 and Bi_T2 groups (Figures 5B,C). However, compared with the Bla_T1 group, subjects in Bla_T2 group had a significantly higher UA and RBC content (Figures 5D,E). The UA content increased from 1800 ± 200 mg/l to 3,500 ± 200 mg/l; the UA content of subject no. 1 increased most drastically, from 1899.4 mg/l to 5330.00 mg/l. The S of the subjects in the Bla group significantly decreased from 8 to 6 h (Figure 5F); these subjects also generally found it difficult to fall asleep. Subjects were assessed for other health indicators as well, namely anorexia, vertigo, acne, memory, anxiety, and constipation. The results are shown in Figure 5G. The subjects in the Bi group were generally in good health, with only a few showing abnormal health conditions. In the Bla group, except for subject no. 5, all had 3–4 abnormal health conditions. Constipation (8 subjects) and anorexia (7 subjects) were common in these subjects, whereas acne was documented less frequently (only 2 subjects). All of the subjects underwent a detailed health investigation before the experiment, which revealed none of the above-mentioned health problems. This indicated that the Bi group subjects adapted better to the CE, whereas the Bla group subjects were easily affected by the CE.

### The differences of gut microbiota lead to different health indicators in confined environment

**Figure 6:**
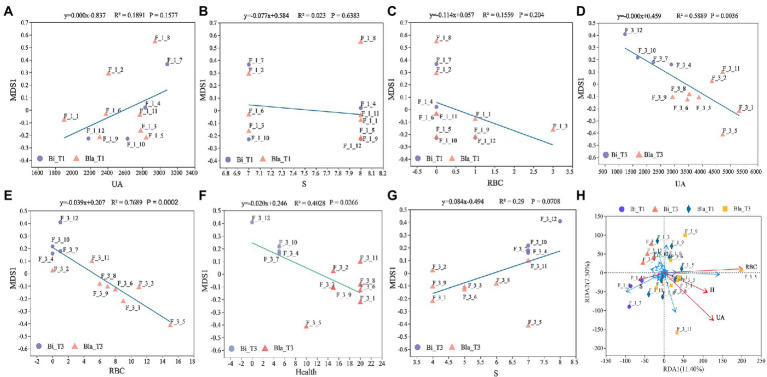
Linear regression analysis of gut microbiota characteristics with different health indicators. **(A)** Linear regression of gut microbiota with UA content at day 1 (T1); **(B)** Linear regression of gut microbiota with sleep time at day 1 (T1); **(C)** Linear regression of gut microbiota with RBC counting at day 1 (T1); **(D)** Linear regression of gut microbiota with UA content at day 14 (T2); **(E)** Linear regression of gut microbiota with RBC counting at day 14 (T2); **(F)** Linear regression of gut microbiota with health conditions at day 14 (T2); Y-axis is the β diversity (NMDS) sorting axis. **(G)** Linear regression of gut microbiota with sleep time at day 14 (T2); **(H)** RDA (Redundancy analysis) of gut microbiota with health status. The length of the health factor arrow can represent the influence of the factor on the gut microbiota. The angles between the arrows represent positive and negative correlations.

Figure 6 shows linear regression analysis of the gut microbiota and UA content, RBC content, S, and other health conditions of the subjects in different groups. There were no significant correlations between the β-diversity of the gut microbiota and health indicators at the beginning of the experiment (day 1, T1 group) (Figures 6A–C). However, the UA content, RBC content, and health indicators were significantly negatively correlated with β-diversity after the experiment (day 14, T2 group, *p* < 0.001) (Figures 6D–F). Although S was positively correlated with β-diversity, this was not significant (Figure 6G, *p* = 0.0708). The RDA analysis was consistent with the ordinal regression results. The UA content, RBC content, and health indicators were significantly correlated with the composition of gut microbiota (*p* < 0.05), and UA content was positively correlated with RBC content and other health indicators. However, there was no significant correlation between S and gut microbiota (*p* = 0.078) (Figure 6H). The UA and RBC content in the Bla group may be correlated with the changes in *Ruminococcus_torques_group, Collinsella*, and other bacteria.

**Figure 7:**
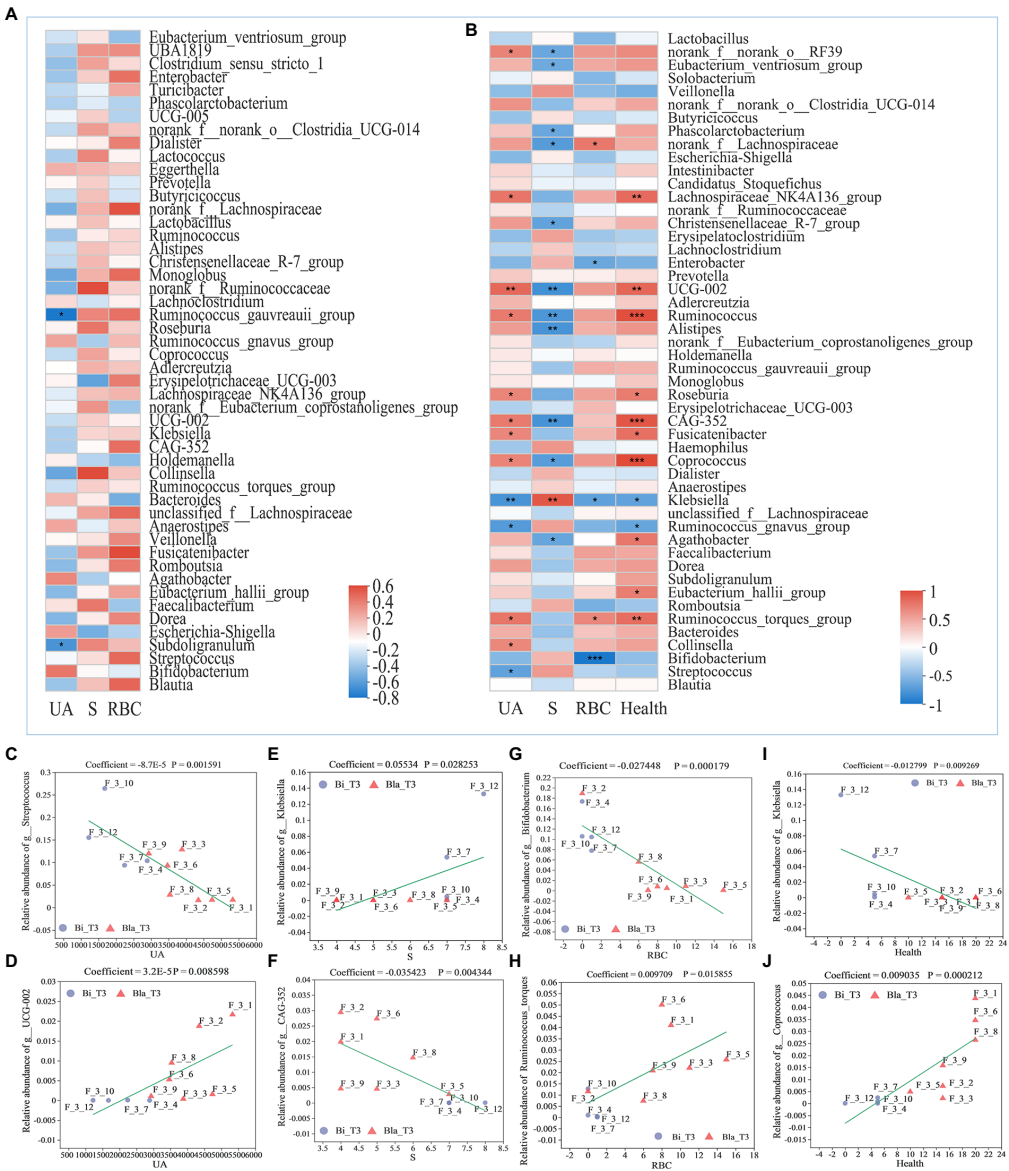
Correlation analysis of gut microbiota composition with different health indicators. **(A)** Correlation analysis between relative abundance of gut microbiota and indicators on genus level at day 1 (T1); **(B)** Correlation analysis between relative abundance of gut microbiota and indicators on genus level at day 14 (T2); **(C)** MaAslin (Multivariate Association with Linear Models) analysis between relative abundance of *Streptococcus* and UA content; **(D)** MaAslin analysis between relative abundance of *UCG-002* and UA content; **(E)** MaAslin analysis between relative abundance of *Klebsiella* and sleep time; **(F)** MaAslin analysis between relative abundance of *CAG-352* and sleep time; **(G)** MaAslin analysis between relative abundance of *Bifidobacterium* and RBC counting; **(H)** MaAslin analysis between relative abundance of *Ruminococcus_torques* and RBC counting; **(I)** MaAslin analysis between relative abundance of *Klebsiella* and health conditions; **(J)** MaAslin analysis between relative abundance of *Coprococcus* and health conditions. *p < 0.05, **p < 0.01, ***p < 0.001.

The Spearman correlation coefficient was used to analyze the correlations between gut microbiota and indexes such as the UA content, RBC content, and health indicators (Figure 7; Boix-Amorós et al., 2016). During the CE experiment, the correlation between the composition of the gut microbiota and health indicators of the subjects changed significantly. The relative abundances of most of the genera were not significantly correlated with health indicators at the beginning of the experiment; only those of *Ruminococcus_gnavus_group* and *Subdoligranulum* were negatively correlated with the UA content (Figure 7A). As shown in Figure 7B, after living for 14 days in the CE, the relative abundances of UCG-002, *Ruminococcus*, *Fusicatenibacter*, *Collinsella*, CAG352, and other genera were significantly positively correlated with the UA content, whereas those of *Klebsiella*, *Ruminococcus_gnavus_group*, and *Streptococcus* were significantly negatively correlated with the UA content. The relative abundance of *Klebsiella* was significantly positively correlated with S, whereas the abundances of CAG-352, UCG-002, *Ruminococcus*, *Alistipes*, and other genera were significantly negatively correlated. The relative abundances of *Bifidobacterium*, *Klebsiella*, and other genera were significantly negatively correlated with the RBC content in urine, whereas those of *Ruminococcus_torques_group* and *norank_f_lachnospiraceae* showed significantly positive correlations. The relative abundances of *Ruminococcus*, CAG-352, *Coprococcus*, and other genera were significantly positively correlated with health indicators, whereas those of *Klebsiella* and *Ruminococcus_gnavus_group* were significantly negatively correlated. The Spearman correlation analysis showed that the changes in CAG-352, *Ruminococcus*, UCG-002, *Klebsiella*, *Ruminococcus_torques_group*, and other genera were consistently correlated with the health indicators of subjects in different groups. The relative abundances of these genera significantly impacted the structure of the gut microbiota.

Multivariate association with linear models (MaAsLin) analysis is a method that investigates the correlations between environmental factors (such as clinical data, physicochemical indicators) and the relative abundance of microbial community species using linear models (Torres et al., 2020). MaAsLin analysis verified that the relative abundances of UCG-002, CAG-352, *Klebsiella*, *Bifidobacterium*, *Coprococcus*, and other genera were significantly correlated with the health conditions of the subjects (Figures 7C–J). The CE experiment showed that, compared with the Bla group, the Bi group was more adaptable to such environments. This difference may be related to the higher relative abundances of *Bifidobacterium*, *Klebsiella*, and other genera and lower relative abundances of UCG-002 and CAG-352 in the Bi group. The high relative abundances of *Ruminococcus_torques_group*, *Ruminococcus*, UGG-002, *Coprococcus*, and other genera in the gut microbiota may make the subjects in the Bla group vulnerable to CEs, thereby increasing their UA content, insomnia, anxiety, and other health abnormalities.

## Discussion

Living in CEs has been linked to issues such as space depression, information isolation, and lack of exercise, which can greatly impact health (Sandal et al., 2006; Arendt, 2012). In addition to special working environments, such as travel and polar exploration, homes have become a relatively common CE due to isolation during the COVID-19 pandemic. In such conditions, the psychological pressure faced by the occupants may lead to metabolic disorders. This can in turn disrupt the bidirectional transmission of the gut–brain axis, causing health problems such as anxiety and circadian rhythm disorders (Risberg et al., 2004; Nie et al., 2020). Most studies of the impact of CEs on health have concentrated on ergonomics, behavior, and other such factors, but not on the composition of gut microbiota. This study investigates the effect of a strict CE (limited living space, information isolation, normal work, and rest time) on the composition of the gut microbiota and health indicators (UA content, sleep time, etc.) and statistically analyzes relevant correlations.

The CE is a complex system with components such as environmental microorganisms, noise, and lighting that have been shown to significantly impact health (Gemignani et al., 2014; Connaboy et al., 2020). We showed that CEs have different effects on the health indicators, such as UA, RBC, and S, of subjects in the Bla and Bi groups. UA (2, 6, 8-trioxypurine) is the end product of purine metabolism (Ridi and Tallima, 2017). However, when the metabolic balance of UA is disrupted, there is excessive retention of UA in the joints and elsewhere in the body; this causes conditions such as gout and diabetes (Vangipurapu et al., 2020). This study shows, for the first time, that CEs significantly increase the UA content in urine, possibly as a result of abnormal metabolism. Studies have shown that UA is a biomarker of the oxidative stress state of the intestinal environment (Wang et al., 2022). Our results indicate that the intestine might be under oxidative stress in people in CEs. In addition to UA, RBCs were abnormally increased in the urine of subjects in the Bla group. There are almost no RBCs in normal urine, but they may enter the urine when glomerular filtration is damaged. This indicates a problem with renal permeability in the Bla group subjects exposed to a CE. Most of the subjects in the Bla group also had insomnia, depression, anorexia, and other health problems. Thus, in a CE, the health indicators of subjects may be closely linked to changes in the gut microbiota through the gut–brain axis (Wei et al., 2022).

The classification of enterotypes provides a reliable framework to understand the microbial diversity of healthy and diseased individuals (Arumugam et al., 2011). The gut microbiota of the subjects was clearly different and could be divided into Bi and Bla groups, and this is not consistent with the traditional enterotype analysis (Falony et al., 2016). However, there are only 12 subjects in this study, the enterotype of people in CE still needs a large number of samples for in-depth analysis. The difference in gut microbiota between enterotypes may affect the metabolic phenotype and responses to diet, pressure, and the external environment, thus affecting human health (Wang et al., 2014; Zhong et al., 2019; Levy et al., 2020).

The gut microbiota not only affect host digestion and metabolic regulation but also modulate the hosts’ mental state and immune function (Jameson and Hsiao, 2018; Simpson et al., 2021; Jose et al., 2022). We found a significant difference in the adaptability of the Bi and Bla groups to a CE. The UA content in the subjects’ urine was positively correlated with the abundances of UCG-002, *Coprococcus*, *Ruminococcus*, *Fusicatenibacter*, and other bacterial genera, which may affect UA metabolism (Chu et al., 2021). Studies have shown significant differences in the composition of gut microbiota between patients with hyperuricemia and healthy individuals, with the imbalance of gut microbiota being related to an increase in UA (Guo et al., 2016; Wei et al., 2022). We found that the relative abundance of *Bifidobacterium* was significantly higher in the Bi group than in the Bla group. Although we did not find a significant association between *Bifidobacterium* and the UA content, other studies have shown that *Bifidobacterium* promotes the degradation of UA in the gut (Guo et al., 2016; Méndez-Salazar et al., 2021). In addition, *Bifidobacterium* has been linked to kidney disease and may reduce the severity of disease in rats (Hanifi et al., 2020; Wang et al., 2020). *Klebsiella*, a commensal genus present in the human gut, has also been detected in CEs such as submarines and the International Space Station (Solomon et al., 2020; Kvesi et al., 2022). We found that *Klebsiella* was significantly enriched in the gut microbiota of the Bi group and negatively correlated with the UA content. Correlation network analysis showed that *Klebsiella* was negatively correlated with *Ruminococcus_torques_group*, *Coprococcus*, and other genera. Thus, it might inhibit the proliferation of related strains and ameliorate gut microbiota disturbances (Oliveira et al., 2020; Flaugnatti et al., 2021).

In our experiments, although the subjects were working and resting normally, their health conditions were affected by the environment; they reported experienced anxiety, memory loss, and anorexia. In the Bla group, *Ruminococcus_torques_group*, *Fusicatenibacter*, *Dorea*, UCG-002, and other genera were significantly enriched and positively correlated with abnormal health indicators. Studies have shown that UCG-002, *Ruminococcus*, and CAG-352 are associated with reduced S (Grosicki et al., 2020). UCG-002 and UCG-003 are the main bacterial genera mediating the positive correlation between chronic insomnia and Coronary Microvascular Dysfunction (CMD; Jiang et al., 2022). Lack of sleep may lead to the accumulation of ROS in intestinal tissue, causing oxidative stress and disrupting the gut microbiota composition (Benedict et al., 2016; Triplett et al., 2020). Additionally, the genera *Fusicatenibacter* and *Dorea* were significantly enriched in the Bla group; this may be associated with decreases in memory or cognitive abilities (Li et al., 2019). Long-term social isolation in CEs could lead to anxiety-like behaviors that impair social relationships and reduce appetite (Donovan et al., 2020). UCG-002 enrichment in the gut of the Bla group has been strongly correlated with reduced appetite (Fluitman et al., 2022).

Recent studies have shown that *Ruminococcus* has an important impact on health (Reau and Suen, 2018). In the Bla group, *Ruminococcus* and *Ruminococcus_torques_group* were significantly enriched and negatively correlated with health indicators such as UA and RBC. Studies have shown that *Ruminococcus* plays an important role in digesting dietary carbohydrates, but that it is also associated with intestinal disorders (irritable bowel syndrome, inflammatory bowel disease, Crohn’s disease, etc.), immune disorders (allergies, eczema, asthma, etc.), and neurological disorders (autism, depression, etc.; Gerber et al., 2013; Hynönen et al., 2016; Crost et al., 2018; Henke et al., 2021). However, *Ruminococcus_gnavus_group* was significantly enriched in the Bi group and negatively correlated with UA, RBC, and other indicators (*p* < 0.05). In germ-free mice, colonization with a single strain of *Ruminococcus_gnavus_group* effectively improved their spatial working memory (Coletto et al., 2022). Chiumento et al. (2019) showed that ruminococcin C1, synthesized by *Ruminococcus gnavus* E1, significantly inhibits the proliferation of pathogenic bacteria in the intestine and alleviates gut microbiota disruption.

The research on the impact of confined space on health is still in a primary stage. This experiment was limited by the experimental conditions. The number of subjects was small and the experiment time was short, so the subjects were mainly subject to short-term stress in CE. The impact of long-term experiment time (>1 month) on health indicators needs to be further studied in CE, especially in the functional analysis of the core gut microbiota. It is expected to improve the health of the people in the CE by regulating the target flora.

## Conclusion

In this study, the effect of a CE on gut microbiota and health conditions were investigated. We documented significant differences in the adaptability of subjects of different enterotypes to the CE. Subjects with the Bi enterotype were more adaptable to the CE than those with the Bla enterotype, who experienced health problems such as elevated UA, lack of sleep, constipation, and abnormal mood. Gut microbiota analysis showed that the compositions of the Bi and Bla enterotypes were significantly different and that the abundances of *Bifidobacterium*, *Dorea*, *Ruminococcus_torques_group*, *Ruminococcus_gnavus_group*, *Klebsiella*, *UCG-002*, *Ruminococcus*, and other genera were significantly associated with health indicators. This study highlights individual differences in the impacts of CEs on human health and the close relationship between the environment and gut microbiota.

## Data availability statement

The data presented in the study are deposited in the BioProject database, accession number PRJNA893603.

## Ethics statement

All experiments were approved and performed following the guidelines of the Ethical Committee of Naval Medical University (Approval No. AF-HEC-018). The patients/participants provided their written informed consent to participate in this study.

## Author contributions

ZC: experiment, investigation, data curation, and writing. ZW: methodology, validation, supervision, reviewing, and editing. DL: methodology, validation, supervision, and project administration. BZ: checked the grammar and revising the article. YX: software, formal analysis, visualization, and writing of the original draft preparation. GW and LA: writing, reviewing, editing, and funding acquisition. CZ and CW: conceptualization, supervision, project administration, and funding acquisition. All authors contributed to the article and approved the submitted version.

## Funding

This work was supported by the Construction of key military disciplines (2020SZ20-3), the Deep Blue Talent Project of Naval Medical University (21TPSL0601), and the National Defense Science and Technology Project (20AH0701, 20AH0702, 20AH0703, and 20AH0704).

## Conflict of interest

The authors declare that the research was conducted in the absence of any commercial or financial relationships that could be construed as a potential conflict of interest.

## Publisher’s note

All claims expressed in this article are solely those of the authors and do not necessarily represent those of their affiliated organizations, or those of the publisher, the editors and the reviewers. Any product that may be evaluated in this article, or claim that may be made by its manufacturer, is not guaranteed or endorsed by the publisher.

## Footnotes

^1^ https://cloud.majorbio.com

^2^ http://drive5.com/uparse/

^3^ http://rdp.cme.msu.edu/
