# Supplementary material for: The gut microbiota as a target to improve health conditions in a confined environment
Source: Front Microbiol. 2022 Dec 19;13:1067756. doi: 10.3389/fmicb.2022.1067756 (PMC9806127; doi:10.3389/fmicb.2022.1067756)
Supplement: Supplementary file 1 [file Table_1.DOCX]

Supplement Table 1 urine physicochemistry

| Subjects | 1 | 2 | 3 | 4 | 5 | 6 | 7 | 8 | 9 | 10 | 11 | 12 |
| --- | --- | --- | --- | --- | --- | --- | --- | --- | --- | --- | --- | --- |
| pH-T1 | 5.000 | 6.000 | 6.500 | 5.500 | 6.000 | 5.000 | 5.500 | 5.000 | 5.000 | 5.000 | 5.500 | 6.000 |
| pH-T2 | 6.500 | 5.500 | 5.000 | 5.500 | 7.000 | 6.500 | 5.500 | 6.000 | 5.500 | 5.000 | 6.000 | 7.000 |
| SG-T1 | 1.015 | 1.018 | 1.020 | 1.029 | 1.024 | 1.020 | 1.020 | 1.027 | 1.025 | 1.025 | 1.024 | 1.014 |
| SG-T2 | 1.027 | 1.023 | 1.021 | 1.025 | 1.016 | 1.024 | 1.023 | 1.013 | 1.029 | 1.020 | 1.020 | 1.011 |
| WBC-T1 | 0.0 | 2.0 | 2.0 | 0 | 3.0 | 14.0 | 1.0 | 0 | 4.0 | 12.0 | 10.0 | 1.0 |
| WBC-T3 | 12.0 | 1.0 | 4.0 | 5.0 | 0 | 10.0 | 1.0 | 7.0 | 8.0 | 3.0 | 1.0 | 0 |
| SQEP-T1 | 0 | 0 | 1 | 0 | 0 | 0 | 0 | 0 | 0 | 1 | 0 | 1 |
| SQEP-T2 | 0 | 1 | 1 | 0 | 0 | 1 | 0 | 0 | 2 | 0 | 0 | 0 |
| LEU-T1 | N | N | N | N | N | N | N | N | N | N | N | N |
| LEU-T2 | N | N | N | N | N | N | N | N | N | N | N | N |
| GLU-T1 | N | N | N | N | N | N | N | N | N | N | N | N |
| GLU-T2 | N | N | N | N | N | N | N | N | N | N | N | N |
| NIT-T1 | N | N | N | N | N | N | N | N | N | N | N | N |
| NIT-T2 | N | N | N | N | N | N | N | N | N | N | N | N |
| PRO-T1 | N | N | N | N | N | N | N | N | N | N | N | N |
| PRO-T2 | N | N | N | N | N | N | N | N | N | N | N | N |
| BIL-T1 | N | N | N | N | N | N | N | N | N | N | N | N |
| BIL-T2 | N | N | N | N | N | N | N | N | N | N | N | N |
| ERY-T1 | N | N | N | N | N | N | N | N | N | N | N | N |
| ERY-T2 | N | N | N | N | N | N | N | N | N | N | N | N |
| ASA-T1 | N | N | N | N | N | N | N | N | N | N | N | N |
| ASA-T2 | N | N | N | N | N | N | N | N | N | N | N | N |
