# Supplementary figures and images for: The gut microbiota as a target to improve health conditions in a confined environment
Source: Front Microbiol. 2022 Dec 19;13:1067756. doi: 10.3389/fmicb.2022.1067756 (PMC9806127; doi:10.3389/fmicb.2022.1067756)

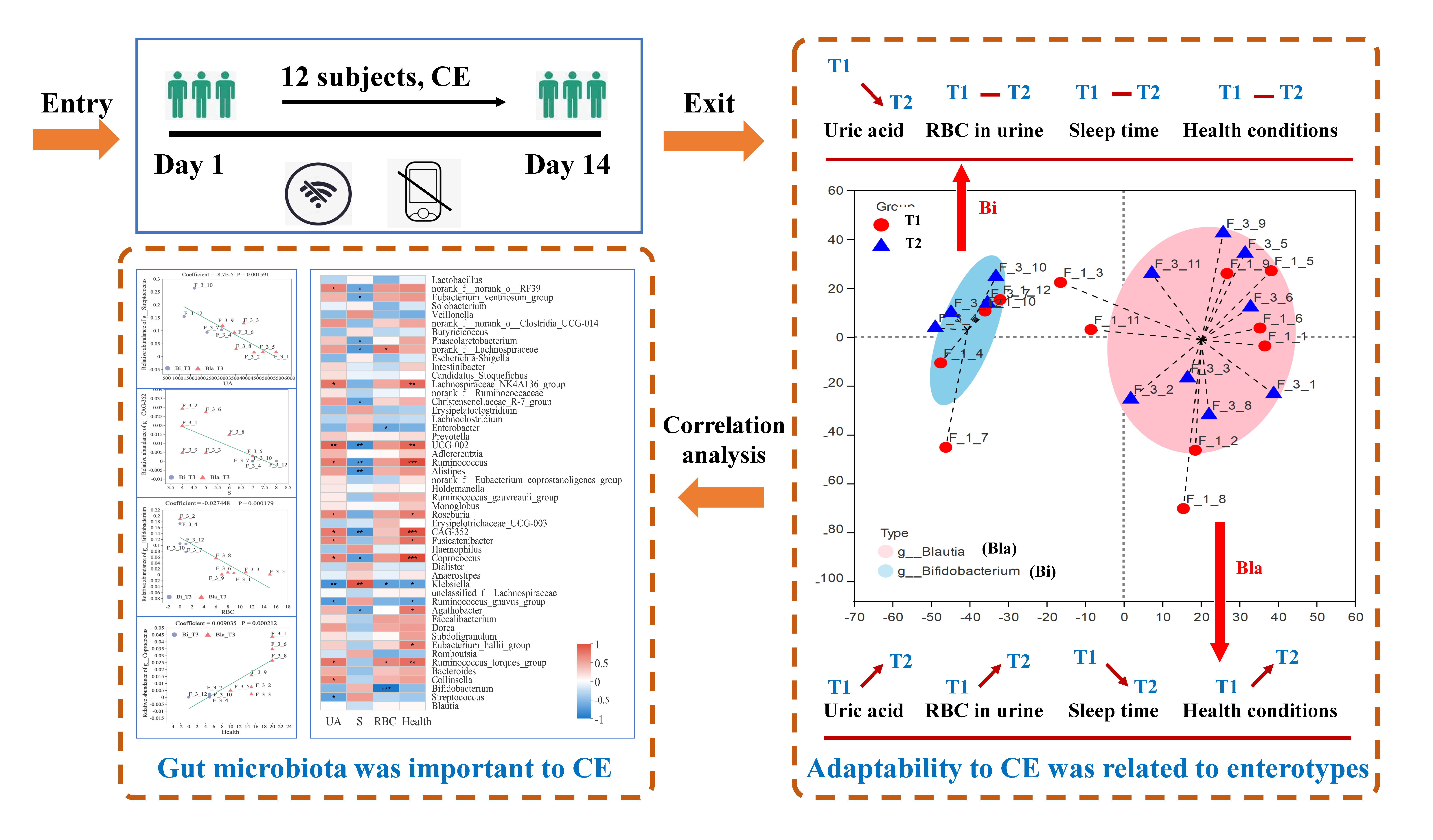

Supplement: Supplementary file 2 [file Image_1.TIF]
